# Community knowledge, attitudes and practices related to schistosomiasis and associated healthcare-seeking behaviours in northern Côte d’Ivoire and southern Mauritania

**DOI:** 10.1186/s40249-018-0453-0

**Published:** 2018-07-10

**Authors:** Amoin Jeanne d’Arc Koffi, Mohamed Doumbia, Gilbert Fokou, Moussa Keita, Brama Koné, N’doumy Noel Abé

**Affiliations:** 1 Department of Research and Development, Centre Suisse de Recherches Scientifiques en Côte d’Ivoire, Abidjan, Côte d’Ivoire; 2 Environment and Communication Training Unit, University Alassane Ouattara, Bouaké, Côte d’Ivoire; 3 Ethno-Sociology Institute, University Félix Houphouët-Boigny, Abidjan, Côte d’Ivoire; 4 Faculty of Letters and Social Sciences, University of Nouakchott, Nouakchott, Mauritania; 5 University Péléforo-Gbon-Coulibaly, Korhogo, Côte d’Ivoire

**Keywords:** Community knowledge, Healthcare-seeking behaviour, Schistosomiasis, Korhogo, Kaédi, Côte d’Ivoire, Mauritania

## Abstract

**Background:**

Among parasitic infections, schistosomiasis ranks second after malaria in terms of worldwide morbidity. Despite efforts to contain transmission, more than 230 million people are infected, of which 85% live in Sub-Saharan Africa. While the epidemiologic characteristics of schistosomiasis have been extensively studied across endemic settings, social factors have been paid less attention. The current study assesses community knowledge of schistosomiasis causes, transmission, signs, symptoms and prevention, as well as healthcare-seeking behaviours in two West African settings, with the aim of strengthening schistosomiasis control interventions.

**Methods:**

From August 2014 to June 2015, we conducted two cross-sectional surveys in Korhogo, Côte d’Ivoire and Kaédi, Mauritania. We applied a questionnaire to collect quantitative data at the household level in Korhogo (*n* = 1456) and Kaédi (*n* = 1453). Focus group discussions (Korhogo: *n* = 32, Kaédi: *n* = 32) and participatory photography (photovoice) (Korhogo: *n* = 16, Kaédi: *n* = 16) were conducted within the communities to gather qualitative data. In addition, semi-structured interviews were used to discuss with key informants from control programmes, non-governmental organizations and health districts (Korhogo: *n* = 8, Kaédi: *n* = 7).

**Results:**

The study demonstrated that schistosomiasis is not well known by the communities; 64.1% claimed to know the causes of the disease, but the reality is different. This knowledge is more from cultural than biomedical source. It was observed that social construction of the disease is different from the biomedical definition. In Korhogo, schistosomiasis was often associated with several other diseases, notably stomach ulcer and gonorrhoea. The populations believe that schistosomiasis is caused by exposure to goat or dog urine in the environment. In Kaédi, schistosomiasis is considered as a disease transmitted by environmenal elements such as sunshine and dirty water. In both settings, the care-seeking pathways were found to be strongly influenced by local customs and self-medication acquired from the informal sector.

**Conclusions:**

This study revealed that knowledge about the aetiology, transmission, symptoms, prevention and treatment of schistosomiasis among the populations in Korhogo and Kaédi is based on their local culture. Deep-rooted habits could therefore pose a significant obstacle to the elimination of schistosomiasis.

**Electronic supplementary material:**

The online version of this article (10.1186/s40249-018-0453-0) contains supplementary material, which is available to authorized users.

## Multilingual abstracts

Please see Additional file 1 for translations of the abstract into the six official working languages of the United Nations.

## Background

In terms of global morbidity, schistosomiasis is the most devastating parasitic disease after malaria. More than 200 million people are currently at risk of contracting schistosomiasis across the globe, 85% of whom live in Africa [1, 2]. In 2016, about 89.2 million individuals have received chemotherapy treatment [3]. It is estimated that across 74 endemic countries the number of annual deaths caused by the disease could be as high as 200 000 [4]. Schistosomiasis, which is caused by different sub-genera of the genus *Schistosoma* – a blood fluke – is responsible for nearly 3.31 million disability-adjusted life years (DALYs) due to anaemia as a result of haematuria, and bladder, kidney, liver and spleen disease [5, 6].

WHO current control strategy of schistosomiasis is based on preventive chemotherapy by periodic administration of the antischistosomal drug praziquantel, particularly, to school age children, considered as high-risk group, alongside with vector control [7]. Although praziquantel reduces morbidity and might impact on transmission, it rarely eliminates infection [8, 9].

Schistosomiasis is an important health concern in West Africa. In Côte d’Ivoire, where it is a major cause of disability and ill health [10], its prevalence ranges from less than 1% to more than 50% depending on the locality [11]. The north of the country has a mean prevalence close to 30%. In Mauritania, transmission of schistosomiasis primarily occurs in the south and south-east, with prevalence rates ranging from 1.3 to 90% [12, 13]. To counteract the persistence of the disease, both the Côte d’Ivoire and Mauritania governments distribute praziquantel and albendazole. The strategy is structured around the involvement of various levels of the health pyramid (i.e. control program, health districts and community health centres). To optimise the disease control practice, health agents are being trained by the national control program in both countries to implement the control protocol more effectively [14, 15].

Despite the widespread prevalence and efforts to contain the disease, it seems that populations of both countries know little about schistosomiasis [16] and health-seeking behaviours are often directed towards traditional methods [17]. Only a few studies have targeted the topic of community knowledge and sub-optimal health-seeking behaviours regarding schistosomiasis epidemiology [16, 17]. However, there is a significant body of evidence that suggests that human behaviours can increase or decrease the risk of schistosomiasis infection [18].

This study aims to assess community knowledge, attitudes and practices (KAP) linked to schistosomiasis in Korhogo, Côte d’Ivoire and Kaédi, Mauritania, in order to determine the impact of knowledge on healthcare-seeking behaviours.

## Methods

### Study area and population

The study was conducted in Korhogo, northern Côte d’Ivoire, and Kaédi, south Mauritania. The choice of those settings is justified by: (i) their respective locations in the southern and northern part of the Sahel band; (ii) Korhogo and Kaédi respectively being located in semi-arid and arid settings, near water bodies (Senegal River for Kaédi and Bandama River for Korhogo); and (iii) the livelihoods of the inhabitants being centred on agriculture, fishing and livestock keeping.

Korhogo is located at 09°27′41” N and 05°38′19” W, and it is the main town of the Poro region. The population of Korhogo town was estimated at 258699 inhabitants in 2014 [19]. The local ethnic group is constituted principally of Sénoufo from the voltaic cultural area. The hydrographical network is dominated by the Bandama River and its tributaries. The annual mean rainfall ranges from 1200 to 1500 mm. The vegetation of the area is west Sudanian Savanna, according to the classification of ecoregions as defined by the World Wide Fund for Nature.

**Fig. 1:**
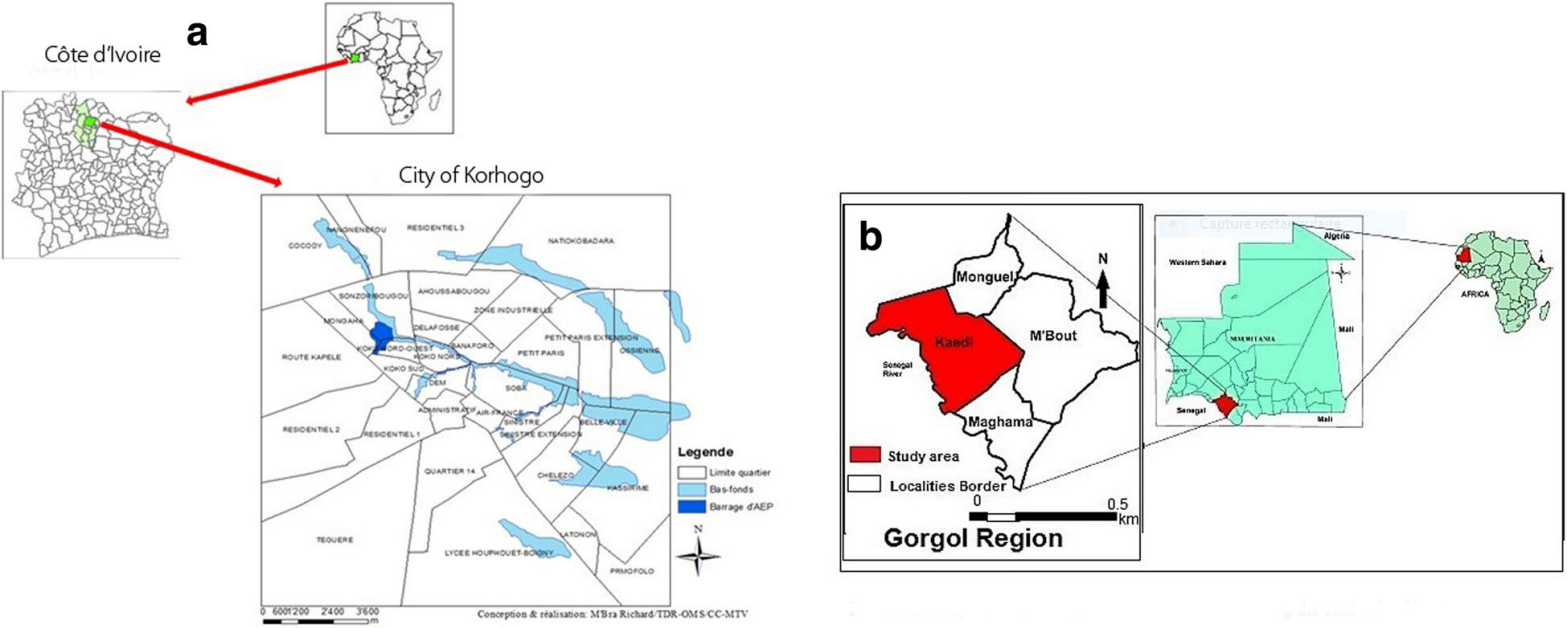
**a**: Map of Republic of Côte d’Ivoire, showing the study site, Korhogo. **b**: Map of Islamic Republic of Mauritania, showing the study site, Kaedi

Kaédi is located at 6°09′02” N and 13°30′ 20” W at the bank of the Senegal River. It is the main town of the Gorgol region, with a (no census data available) population estimated at 121000 inhabitants in 2013 [20, 21]. The Kaédi population is composed of several ethnic groups, namely Halpulaar, Moors, Soninké and Wolof. The mean annual rainfall is 300 to 500 mm and the vegetation is of the Sahelo-Sudanese type. From the livelihood activities carried out including agriculture and fishing, Kaédi appears to be the main breadbasket of Mauritania (see Fig. 1).

### Study procedures

Two cross-sectional surveys were conducted in each setting, one during the dry season and one during the rainy season. Prior to the study’s inception, both administrative and local authorities were informed about the study’s aims, procedures, benefits and eventual risks. The study was designed combining qualitative and quantitative approaches. Data collected with qualitative methods (i.e. observations and interviews) were triangulated with household questionnaires and literature review.

For the quantitative approach, 1456 and 1453 households were sampled in Korhogo and Kaédi, respectively. The survey team used a questionnaire and visited each selected household unit to collect data on KAP pertaining to schistosomiasis. The household head or his representative was interviewed by investigators who were trained in data collecting techniques in order to reduce biases.

For the qualitative approach, 32 focus group discussions (FGDs) were conducted in each community, 16 participatory photography exercises (photovoice) and 15 semi-structured interviews were also conducted in both countries (8 in Korhogo and 7 in Kaédi) either with community members or key informants from the national control program, non-governmental organisations (NGOs) and health districts. Data collection was conducted in the local languages with support from local trained field assistants. The survey was conducted in the Senoufo language in Korhogo (the most widely spoken language in the area), while Pulaar and Hassania arabic, the most popular languages of the Gorgol region, were alternatively used for data collection in Kaédi. Any potential bias was reduced by training the assistants for two days on basic concepts and notions related to the study and data collection methodology. The responses were firstly recorded in local languages and then translated into French during systematic transcription using Microsoft Word 2013 (Microsoft Corporation, Redmond, WA, USA).

The FGDs were conducted separately for different sex and age groups, resulting in four groups constituted of young men, young women, adult men and adult women. Potential participants were screened for eligibility. The selected ones were those who have lived in the community at least six months prior to the interview to avoid selection bias. Male and female participants aged 18–35 were selected for the age category of ‘young’ and those aged from 36 were selected for the age category of ‘adult’. The choice of these categories was motivated by the fact that perceptions at the community level vary according to social positions and status that are basically defined by age and sex. Participants must have also lived in the study area for more than six months prior to the start of the survey and able to communicate in the local language. At the community level, permission was obtained from the local authorities following a briefing on the study. A contact person who facilitated the introduction to the village and assisted in mobilising each category was chosen by the community leader. The number of participants in each focus group varied from seven to 10 for each of the four groups per study site. For each study site, 16 FGDs were conducted during each season (rainy and dry) resulting in a total of 32 FDGs per site.

Participatory photography or photovoice consisted of 16 community members taking photographic images in each study location. Selected individuals in some households were given a disposable camera and asked to take photos of everything they thought was related to schistosomiasis. It was explained to them that the photographs should be made in strict respect of people’s privacy and that the images should not allow for the recognition of individuals or their property. Photographers were then interviewed individually and collectively about the content and meaning of their photos, as well as the messages they wanted to convey through the photos. This was done following an interview protocol summarised under the acronym SHOWeD: ‘what we **S**ee – what is **H**appening – relation to **O**ur lives – **W**hy does this exist – what to **D**o about it’ [22]. Photos were then analysed individually and collectively to understand the knowledge of communities related to schistosomiasis and the strategies put in place to control the disease.

Semi-structured interviews were also conducted with individual key informants both in the population and in health facilities, using an interview guide, with the aim of noting down not only the habits and ideologies of the populations, but also evaluating the local policies and care and control strategies. The key persons involved were health facility workers, municipal and traditional authorities, and local development NGOs.

### Sample size

The questionnaire for eliciting complementary quantitative data was applied in 1456 households in Korhogo and 1453 in Kaédi. The number of households was selected randomly from each town. The sample size *N* was calculated using the following formula [21, 23]:

*N* = δ^2 × *p*(1-*p*) × c)/i^2,

where δ = 1.96, *P* = 0.35, expected prevalence of schistosomiasis; c = 2, correction factor; and *i* = 0.05, margin of error.

### Data analyses

Quantitative data were entered into EpiData version 3.5.3 (The EpiData Association, Odense, Denmark) and analysed using SPSS version 18 (IBM Corp, Chicago, USA). General community knowledge of schistosomiasis and healthcare-seeking behaviours regarding schistosomiasis were analysed and expressed in proportions. The chi-square test and Fisher’s exact test were used to show correlations between variables.

Qualitative data were processed using MaxQDA version 12 (VERBI GmbH, Berlin, Germany). Data recorded during interviews were systematically transcribed into Microsoft Word (Microsoft Corporation, Redmond, WA, USA). Information on transcribed text was then grouped according to pre-established codes based on the interview guide and key covariates used for the study. After the first coding, data were recoded for further content analysis.

## Results

### Socio-demographic characteristics of informants

The proportion of male to female respondents was 45.5 to 54.5% in Kaédi and 45.5 to 54.5% in Korhogo, respectively. A significant number of participants were living under monogamous marriage regime (65.6% in Kaédi and 49.1% in Korhogo), and most were Muslims (98% in Kaédi and 71.5% in Korhogo). The majority of the participants worked in the informal sector comprising traders, artisans, farmers, herdsmen and intermediaries (62.3% in Kaédi and 68.4% in Korhogo). About a third of the respondents in both sites have never received any formal education (32.7% Kaédi and 39.8% Korhogo). About a third of respondents from Kaédi (32.6%) have received Arabic education in Coranic school, while about a quarter in Korhogo had a secondary school level of education (23.7%) (Additional file 2: Table S1 summarizes the socio-demographic characteristic).

### Knowledge of schistosomiasis

#### Local names of schistosomiasis

The local names of schistosomiasis in Korhogo and Kaédi are summarized in Table 1. The identified entities with respect to schistosomiasis were different from one site to another. In Korhogo, four different names for schistosomiasis were reported – linked to the urogenital system and gastrointestinal tract. Two of the local names, ‘*sonfichichan*’ and ‘*firmaning*’, designate urinary pain; ‘*sonfichichan*’ refers to the pain during urination, while ‘*firmaning*’ is translated as the ‘root of urine’. Thus, any infection related to the genital tract and any disease that pertains to sex is referred to as the ‘root of urine’. However, after the clinical signs of schistosomiasis were described to the participants, it became clear that ‘*firmaning*’ was not directly related to the infection but referred to the inflammation of the prostate or gonorrhoea.

**Table 1:** Local names for schistosomiasis according to locality and ethnic group

Ethnic group	Type of schistosomiasis	Local name	Meaning of local name
Korhogo			
Senoufo	Black schistosomiasis	Firmaning	Schistosomiasis due to a curse (pain during urination, blood after urination)
	White schistosomiasis	Firmaning	Schistosomiasis due to a contact with an infected person
		Sonfichichan	Difficulty to urinate
	Intestinal schistosomiasis	Tôtônou or lagbô	Another form of white schistosomiasis (pain during urination)
Malinké	Intestinal schistosomiasis	Tôtônou	Glow in stool
Kaédi			
Halpulaar	Black schistosomiasis	Boobri balleri	Schistosomiasis due to water
	White schistosomiasis	Boobri doneri	Schistosomiasis due to heat
Moors	White schistosomiasis	Issri lébiane	Schistosomiasis due to heat
	Black schistosomiasis	Issri lékal	Schistosomiasis due to urination on someone’s else urine
	Pain during urination	Issri bolt	Schistosomiasis due to heat

Intestinal schistosomiasis is often confused with dysentery, that is named ‘*lagbô*’ in Senoufo and ‘*tôtônou’*, a name borrowed from their Malinké neighbours (Malinké are a neighbouring ethnic group to the Senoufo in north Côte d’Ivoire sharing the same livelihood activities and habits). These two expressions are descriptive of diarrhoea with blood and mucus in the stool, that is meant to describe dysentery. However, owing to the similarity of symptoms between both diseases and a lack of information, these two terms are also applied to describe schistosomiasis.

In Kaédi, there is a single name to designate schistosomiasis among each ethnic group. Participants were able to provide some of the local names from the community that were associated with symptoms and causes of schistosomiasis. The Halpulaar use the word ‘*boobri*’ for the disease while the Moors call it ‘*issri bolt*’*.* These local names referred to elements of the natural environment, such as water and sun, for both ethnic groups. According to both groups, schistosomiasis can be contracted through water or sun, thus there is a name to designate schistosomiasis that is contracted by sun and another by water. Among the Moors, it is believed that one can contract schistosomiasis by urinating on someone’s else urine. In both groups, schistosomiasis due to water was perceived as the most complicated.

#### Social aetiology of schistosomiasis

In general, the causes of schistosomiasis mentioned by the study participants varied between the study sites and ethnic groups. From the Senoufo’s perspective, the disease is linked to the environment and to mysticism. In relation to the environment, it is believed that there are several transmission routes: (i) stepping on urine from goats or dogs; (ii) drinking unsafe water or playing in dirty water; and (iii) using dirty and defected toilets. This last point was illustrated in Korhogo, where a participant described one infection route as follows:

‘*I have been a victim of schistosomiasis we are talking about*. *One can contract it in the toilet. For example, if you urinate where someone who is infected has just urinated, you can be contaminated.*’ (FGD with adult women, Korhogo, April 2015).

According to this perspective, a lack of hygiene and sanitation is considered as an enabling factor for the occurrence of schistosomiasis. However, mystic causalities are also blamed. They are referred to in particular when a violation of social rules is involved, such as committing adultery. According to a traditional healer:

‘*When men commit adultery, this can cast a curse on them. They are going to get infected.*’ (Interview with a traditional healer, Korhogo, April 2015).

In Kaédi, two causes for schistosomiasis were identified: environmental causes and natural causes. According to Halpulaar, schistosomiasis is transmitted through water contact during activities related to water, or when consuming either unsafe water or water heated by the sun. The Halpulaars link the disease to water because their village was formerly surrounded by water and the disease was rampant. Thus, they have been told that water was the cause of the disease. Among the natural causes, the Moors believe that schistosomiasis is caused by sunrays and by walking barefoot on hot sand.

#### Knowledge of disease symptoms

Knowledge of the symptoms and manifestations of schistosomiasis is summarized in Table 2. Overall, 82.7% of respondents identified the disease by blood in urine, 34.9% by low urine output and 33.6% by pain during urination. The most commonly mentioned symptom across both settings was blood in urine (69.2% in Korhogo and 86.6% in Kaédi) and pain during urination (28.8% in Korhogo and 34.9% in Kaédi). Low urine output was mentioned more often in Kaédi as compared with Korhogo (44.0% versus 6.8%, respectively).

**Table 2:** Knowledge of schistosomiasis symptoms according to locality

	Kaédi *n* = 503		Korhogo *n* = 195		Total *n* = 698	
	Yes (*n*)	Yes (%)	Yes (*n*)	Yes (%)	Yes (*n*)	Yes (%)
*Do you know about schistosomiasis symptoms?*	441	87.7	143	73.3	584	83.7
Pain during urination	154	34.9	42	28.8	196	33.6
Low urine output	194	44.0	10	6.8	204	34.9
Blood in stool	204	46.3	72	49.3	276	47.3
Stomach pain	93	21.1	8	5.5	101	17.3
Headache	61	13.8	7	4.8	68	11.6
Blood in urine	382	86.6	101	69.2	483	82.7
^a^Other symptoms	14	3.2	25	17.1	39	6.7

^a^Wound on genitals, constipation; *n*: Number of respondents

It was found that in Korhogo the level of education does not influence the knowledge of schistosomiasis symptoms (see Table 3). However, in Kaédi the level of education seemed to be relevant for the identification of symptoms such as low urine output and frequent urination (65.0% of responses, *P* = 0.001). However, symptoms such as pain during urination seemed to be more known by people who had a higher level of education (43.0% of responses). Blood in urine was a well-known symptom by all respondents regardless of their level of education in both localities (> 80%).

**Table 3:** Population’s knowledge of schistosomiasis symptoms according to level of literacy in Korhogo and Kaédi

	No education (%)	Arabic (%)	Primary (%)	Secondary (%)	High school (%)	*P*-value
Korhogo	*n* = 576	*n* = 155	*n* = 263	*n* = 343	*n* = 107	
*Knowledge of schistosomiasis*	54 (9.4)	17 (11)	49 (18.6)	106 (30.6)	45 (42.1)	**≤ 0.001** ^**a**^
Pain during urination	9 (39.1)	6 (54.4)	9 (37.5)	14 (24.6)	4 (0.05)	0.5^b^
Low urine output	3 (13.0)	1 (9.1)	3 (12.5)	3 (5.3)	0 (0.0)	0.291^b^
Blood in stool	11 (47.8)	4 (36.4)	8 (33.3)	31 (54.4)	16 (55.2)	0.384^a^
Stomach pain	0 (0.0)	1 (9.1)	2 (8.2)	3 (5.3)	1 (3.4)	0.668^b^
Headache	0 (0.0)	0 (0.0)	1 (4.2)	4 (7.0)	2 (6.9)	0.628^b^
Blood in urine	11 (47.8)	9 (81.8)	19 (79.2)	39 (68.4)	22 (75.9)	0.111^a^
^c^Other symptoms	7 (30.4)	2 (18.2)	1 (4.2)	8 (14.0)	7 (24.1)	0.135^b^
Kaédi	*n* = 473	*n* = 472	*n* = 236	*n* = 195	*n* = 72	
*Knowledge of schistosomiasis*	276 (58.4)	228 (48.3)	122 (51.7)	107 (54.9)	35 (48.6)	**0.031** ^**a**^
Pain during urination	52 (30.6)	49 (43.0)	24 (35.8)	18 (27.3)	10 (43.5)	0.129^a^
Low urine output	66 (38.8)	65 (57.0)	26 (38.8)	21 (31.8)	15 (65.2)	**0.001** ^**a**^
Blood in stool	76 (44.7)	56 (49.1)	29 (43.3)	29 (43.9)	13 (56.5)	0.755^a^
Stomach pain	35 (20.6)	30 (26.3)	12 (17.9)	8 (12.1)	7 (30.4)	0.151^a^
Headache	28 (16.5)	22 (19.3)	6 (9.0)	2 (3.0)	3 (13.0)	**0.020** ^**b**^
Blood in urine	144 (84.7)	98 (86.0)	61 (91.0)	61 (92.4)	17 (73.9)	0.146^a^
^c^Other symptoms	8 (4.7)	3 (2.6)	3 (4.5)	0 (0.0)	0 (0.0)	0.321^b^

Significant *P*-values (< 0.05) are highlighted in bold

^a^*P*-value based on chi-square test; ^b^*P*-value based on Fisher’s exact test; ^c^Wound on the reproductive organs; *n*: Number of respondents

#### Knowledge of transmission pathways

Regarding schistosomiasis transmission, 63.8 and 65.1% of participants in Kaédi and Korhogo, respectively, affirmed knowing the route (see Table 4). However, 62.5% of respondents in Kaédi and 37.9% in Korhogo stated that drinking unsafe water transmits schistosomiasis. Furthermore, contact with dirty water was mentioned as a source of infection (50.7% in Kaédi and 42.6% in Korhogo). Swimming was cited as another way of becoming infected by schistosomiasis (59.1% in Kaédi and 65.3% in Korhogo). In fact, in Kaédi, knowledge of schistosomiasis transmission pathway is linked to education level. For instance, swimming in the river is more cited by educated people compared to non-educated ones (*P* <  0.001) etc. (see Table 5).

**Table 4:** Knowledge of transmission pathway of schistosomiasis according to locality

	Kaédi *n* = 779		Korhogo *n* = 289		Total *n* = 1068	
	Yes (*n*)	Yes (%)	Yes (*n*)	Yes (%)	Yes (*n*)	Yes (%)
*Do you know how schistosomiasis is transmitted?*	497	63.8	188	65.1	685	64.1
Drinking unsafe water	312	62.5	72	37.9	384	56.1
Eating poorly washed fruits, vegetables	81	16.2	5	2.6	86	22.4
Swimming in rivers, drains, etc.	295	59.1	124	65.3	419	61.2
Contact with infected person	41	8.2	15	7.9	56	13.4
Walking barefoot on infected urine	110	22.0	13	6.8	123	18.0
Contact with dirty water	253	50.7	81	42.6	334	48.8
^a^Other transmission	38	7.6	22	11.6	60	18.0
Do not know	6	1.2	2	1.1	8	13.3

*n*: Number of respondents; ^a^Avoiding contact with goat urine, wearing protective shoes, avoiding drinking hot water

**Table 5:** Knowledge of transmission pathways of schistosomiasis according to level of literacy in Korhogo and Kaédi

	No education (%)	Arabic (%)	Primary (%)	Secondary (%)	High school (%)	*P*-value
Korhogo	***n*** **= 26**	***n*** **= 13**	***n*** **= 33**	***n*** **= 75**	***n*** **= 38**	
Drinking unsafe water	8 (29.6)	5 (38.5)	11 (34.4)	32 (42.1)	15 (39.5)	0.817^b^
Eating poorly washed fruits, vegetables	1 (3.7)	1 (7.7)	0 (0.0)	2 (2.6)	1 (2.6)	0.692^b^
Swimming in rivers, drains, etc.	11 (40.7)	7 (53.8)	25 (78.1)	51 (67.1)	26 (68.4)	0.035^a^
Contact with infected person	4 (14.8)	3 (23.1)	1 (3.1)	4 (5.3)	1 (2.6)	**0.042** ^b^
Walking barefoot on infected urine	3 (11.1)	1 (7.7)	1 (3.1)	8 (10.59	N/A	0.217^b^
Contact with dirty water	11 (40.7)	9 (69.2)	10 (31.3)	35 (46.1)	15 (39.5)	0.200^a^
^c^Other	6 (22.2)	2 (15.4)	2 (6.3)	10 (13.2)	2 (5.3)	0.230^b^
Do not know	1 (3.7)	0 (0.0)	0 (0.0)	0 (0.0)	1 (2.6)	0.415^b^
Kaédi	***n*** **= 189**	***n*** **= 136**	***n*** **= 86**	***n*** **= 76**	***n*** **= 27**	
Drinking unsafe water	137 (72.5)	76 (60.3)	44 (55.0)	36 (47.4)	18 (66.7)	**0.001** ^**a**^
Eating poorly washed fruits, vegetables	26 (13.8)	29 (23.0)	13 (16.3)	7 (9.2)	6 (22.2)	0.073^a^
Swimming in rivers, drains, etc.	79 (41.8)	82 (65.1)	56 (70.0)	59 (77.6)	18 (66.7)	**< 0.001** ^a^
Contact with infected person	9 (4.8)	13 (10.3)	9 (11.3)	5 (6.6)	5 (19.2)	0.058^a^
Walking barefoot on infected urine	45 (23.8)	31 (24.6)	13 (16.3)	9 (11.8)	12 (44.4)	**0.005** ^**a**^
Contact with dirty water	85 (45.0)	73 (57.9)	42 (52.5)	36 (47.4)	16 (59.3)	0.175^a^
^c^Other	17 (9.0)	13 (10.3)	3 (3.8)	3 (3.9)	2 (7.4)	0.291^b^
Do not know	3 (1.6)	1 (0.8)	0 (0.0)	2 (2.6)	0 (0.0)	0.555^b^

Statistically significant *P*-values (< 0.05) are highlighted in bold

*n*: Number of respondents; ^a^*P*-value based on chi-square test; ^b^*P*-value based on Fisher’s exact test; ^c^Contact with goat urine, not wearing protective shoes, drinking hot water, exposure to sunlight

During the FGDs, behaviours such as drinking unsafe water, swimming and walking under the sun were mentioned as risk factors for infection.

#### Knowledge of prevention measures

In Kaédi, among the respondents who were aware of schistosomiasis, less than half cited at least one effective protective measure (see Table 6). Only 30% of all the respondents (*n* = 1450) stated that they knew what the preventive behaviours for schistosomiasis are. In Korhogo, respondents cited that avoiding swimming is a unique effective preventive measure (75.9%).

**Table 6:** Knowledge of schistosomiasis prevention measures according to locality

	Kaédi *n* = 1450		Korhogo *n* = 1443		Total *n* = 2893	
	Yes (*n*)	Yes (%)	Yes (*n*)	Yes (%)	Yes (*n*)	Yes (%)
*Do you know how to avoid schistosomiasis?*	438	30.2	173	12.0	611	21.1
Avoid eating certain foods	77	17.5	4	2.3	81	13.3
Avoid drinking unsafe water	285	65.1	86	49.4	371	60.7
Avoid swimming in rivers, drains, etc.	214	48.9	132	75.9	346	56.3
Avoid urinating in water bodies	198	45.2	37	21.3	235	37.9
Avoid defecating in water bodies	201	45.9	31	17.8	232	37.9
^a^Other	25	5.7	24	13.8	49	21.1
Do not know	6	1.4	2	1.1	8	16.3

*n*: Number of respondents; ^a^Avoid contact with goat urine, wear protective shoes, avoid drinking hot water

Table 7 summarizes the participants’ knowledge of preventive means for schistosomiasis control. In Korhogo, the level of education was found to be correlated with the knowledge of the control measure, such as swimming. The frequencies of responses were higher in the highest educated groups compared with the lowest educated groups (78.8–88.2% versus 42.9–47.8%; *P* <  0.001*).* Other risk factors, such as avoiding urinating and defecating in water, were little known, regardless of a person’s education level. The impact of the education level on the knowledge of prevention measures in Korhogo was weak or not clearly demonstrated. In Kaédi, among the prevention measures, avoiding drinking unsafe water and avoiding swimming were often mentioned. In fact, 77.0% of non-educated people and 50.0% of educated respondents mentioned that drinking unsafe water should be avoided. 70.4% of respondents with a higher education mentioned avoiding swimming as a protective measure compared with 31% of the non-educated individuals (*P* <  0.00).

**Table 7:** Knowledge of schistosomiasis prevention measures according to level of literacy in Korhogo and Kaédi

	No education (%)	Arabic (%)	Primary (%)	Secondary (%)	High school (%)	*P*-value
Korhogo	***n*** **= 23**	***n*** **= 14**	***n*** **= 34**	***n*** **= 68**	***n*** **= 33**	
Avoid eating certain foods	0 (0.0)	2 (14.3)	1 (2.9)	1 (1.5)	0 (0.0)	**0.033** ^**b**^
Avoid drinking unsafe water	9 (39.1)	5 (35.7)	16 (47.1)	39 (57.4)	16 (48.5)	0.430^b^
Avoid swimming in rivers, drains, etc.	11 (47.8)	6 (42.9)	27 (79.4)	60 (88.2)	26 (78.8)	**< 0.001** ^**a**^
Avoid urinating in water bodies	6 (26.1)	4 (28.6)	3 (8.8)	17 (25.0)	6 (18.2)	0.319^b^
Avoid defecating in water bodies	3 (13.0)	1 (7.1)	2 (5.9)	18 (26.5)	6 (18.2)	0.079^b^
^a^Other	5 (21.7)	5 (35.7)	3 (8.8)	7 (10.3)	4 (12.1)	0.082^b^
Do not know	0 (0.0)	0 (0.0)	0 (0.0)	2 (2.9)	0 (0.0)	0.542^b^
Kaédi	***n*** **= 174**	***n*** **= 106**	***n*** **= 63**	***n*** **= 72**	***n*** **= 24**	
Avoid eating certain foods	30 (17.2)	25 (23.6)	11 (17.5)	5 (6.9)	6 (25.0)	0.056^b^
Avoid drinking unsafe water	135 (77.6)	63 (59.4)	35 (55.6)	36 (50.7)	16 (66.7)	**< 0.001** ^**a**^
Avoid swimming in rivers, drains, etc.	54 (31.0)	58 (54.7)	38 (60.3)	50 (70.4)	14 (58.3)	**< 0.001** ^**a**^
Avoid urinating in water bodies	76 (43.7)	60 (56.6)	24 (38.1)	26 (36.6)	12 (50.0)	0.053^a^
Avoid defecating in water bodies	77 (44.3)	60 (56.6)	25 (39.7)	27 (38.0)	12 (50.0)	0.092^a^
^c^Other	8 (4.6)	8 (7.5)	5 (7.9)	2 (2.8)	2 (8.3)	0.548^b^
Do not know	2 (1.2)	2 (1.9)	2 (3.2)	0 (0.0)	0 (0.0)	0.541^b^

Statistically significant *P*-values (< 0.05) are highlighted in bold

n: number of respondents; ^a^*P*-value based on chi-square test; ^b^*P*-value based on Fisher’s exact test; ^c^avoiding contact with goat urine, wearing protective shoes, avoiding drinking hot water

The choice of prevention measures depends on people’s perceptions with regards to the disease. In fact, the Halpulaar believe that not consuming unsafe water makes it possible to avoid schistosomiasis. The Moors believe that the disease is preventable by no exposure to sunrays. They recommend that:

‘*to avoid the disease (schistosomiasis), one should not drink dirty water or water that has been heated by the sun*’ (FGD with women, Kaédi, June 2015).

It appears that, the local populations have poor knowledge of prevention means.

### Impact of knowledge of the disease on healthcare-seeking behaviours

Care practices varied among ethnic groups. It appeared that the communities’ knowledge of the disease socially constructs people’s care-seeking choices. Praziquantel is an effective treatment against all human forms of schistosomiasis. However, according to the populations’ knowledge of the disease, their healthcare-seeking behaviours differ from curative solutions proposed by medical science.

In Korhogo, respondents believe there are two kinds of schistosomiasis: natural and mystic. The natural form as designated by community is the same as the one defined by biomedical medicine, and is also acquired through walking on goat or dog urine. However, the mystic form is believed to be contracted through witchcraft. Care-seeking behaviours depend on those perceptions. In Kaédi, the population recognises the natural form of schistosomiasis. However, the perception around the transmission pathway (i.e. exposure to sunlight, drinking hot water) influences care-seeking behaviours and leads to the use of inappropriate ways of protection.

#### Treating the natural form of schistosomiasis

To treat the natural form, people rely firstly on self-medication, local customs and drugs sourced from the informal sector (e.g. street markets). Going to the hospital appears to be the last resort. A local custom involves a stone being heated, which the patient crouches over, and the heat that emerges from it goes towards the genitals and cures the disease. Beyond this practice, communities self-medicate often using a black powder of unknown composition or a capsule commonly called *‘toupaille’* that is mixed with a soft drink, both of which are sold in the informal sector.

It appears that cultural conceptions of schistosomiasis influence health-seeking behaviours, as evidenced by the following statement:

*‘Schistosomiasis is rare, so we do not know how to treat ourselves.*’ (FGD with adult women, Korhogo, April 2015).

In addition, various avenues for seeking treatment were pointed out including: traditional healers, hospitals and street healers. An adult female participant stated:

‘*I was once suffering from schistosomiasis, so I did the indigenous treatment. It did not work, then I went to the American dispensary. They examined me, they took my urine and they found it was this disease. So they gave me a treatment that I took, and then I was also given an appointment, so when I went back there, they gave me the rest of the medication and I was cured.*’ (FGD with adult women, Koko, Korhogo, April 2015).

Participants agreed that they do not know the effective treatment. One stated:

‘*One of my nieces has been infected with this disease. So really, we did everything, it did not work, there’s black powder medicine there, at home. They used that, it did not work*.’ (FDG with young women, Korhogo, April 2015).

#### Treating the mystic form of schistosomiasis

According to participants, the mystic form of schistosomiasis can only be treated by traditional healers through prayers and medicinal plants. In Kaédi, most participants agreed that schistosomiasis is curable. One avenue for seeking treatment that was pointed out by both ethnic groups was domestic processes. Care is strongly based upon self-medication: Moors use ‘*hénné*’: bathing with cold water to lower the body temperature. According to the respondents, schistosomiasis is present when the body is hot because of exposure to sunrays. They believe that the heat generated by the sun is accumulated in the lower abdomen and the burning causes schistosomiasis.

The Halpulaar use a powder made from a tree called ‘*tékié*’. This powder is mixed with water or milk and ingested as a therapeutic drink. They also use a plant called ‘*N’nan*’ for treatment, which is also mixed with milk.

Data from both sites revealed that there is no effective means to prevent schistosomiasis and, at the household level, there are no particular practices to control schistosomiasis.

## Discussion

Regardless of locality, study participants had similar levels of KAP relating to schistosomiasis control. However, it seems that communities in Kaédi had more knowledge about schistosomiasis signs and symptoms compared with their counterparts in Korhogo.

Generally, the results around people’s knowledge in both settings are in line with those from studies conducted in Ethiopia, Ghana and Côte d’Ivoire; these focused on perceptions of the general population and found low levels of knowledge about the disease even in endemic areas [17, 21, 24]. However studies in other schistosomiasis-endemic areas have found a general high level of awareness of schistosomiasis. A study conducted in Zimbabwe, for instance, reported that 80% of people in villages were aware of schistosomiasis [25]. Similarly, a study conducted in Brazil revealed that people were fairly familiar with schistosomiasis [26]. Findings from a study in Kenya also showed that schistosomiasis is known by populations, but its recognition as a major health concern is still limited among the communities assessed [23]. The low level of knowledge in Korhogo and Kaédi regarding schistosomiasis is explained by changes in environmental conditions (relocation, building of agricultural dams). In Korhogo, the building of dams in recent years for agricultural and pastoral activities throughout the seasons has contributed to rapidly changing environmental conditions, what has increased schistosomiasis prevalence [27]. In Kaédi, the Moors ethnic group did not link the epidemiology of schistosomiasis to water contact because they originate from an area of Mauritania where access to water is limited. Even among those who have moved to new residential places near water points (Senegal River) generations ago, the level of knowledge of the disease has not increased. In addition, the lack of sensitisation concerning the disease contributed to the communities’ ignorance, thus making them vulnerable to the disease [28, 29].

A variety of local names designate schistosomiasis disease, such as ‘*sonfichichan*’, ‘*firmaning*’ (also used for gonorrhoea) and ‘*lagbô*’ (intestinal schistosomiasis). The disease is often confused with dysentery or stomach ulcer in Korhogo. In fact, a study conducted by Acka and colleagues in the endemic area of Man in western Côte d’Ivoire in 2010 highlighted the confusion concerning the designation of the disease [17]. In Kaédi, ‘*boobri*’ and ‘*issri bolt*’ mean urinary schistosomiasis among the Halpulaar and Moors, respectively. Local names connote the disease’s signs and symptoms. The concept of naming a disease through signs and symptoms was also reported by Biays and colleagues in Cambodia where people used a variety of names to designate schistosomiasis in the Khmer language; ‘*santéas omal*’ means ‘the disease of the big bellies’; ‘*dam ksir*’ translates word by word as ‘crab pipe’ and refers to abdominal pains like the sensation of crab claws. The expression ‘*tleak andaek*’ illustrates splenomegaly by the ‘falling turtle’ in the abdomen, while ‘*teach tuk*’ (‘water in the belly’) is the name given to ascites [30].

In general, the proportion of respondents who mentioned different disease symptoms in Kaédi was higher compared with those in Korhogo. This could be explained by the fact that people in Kaédi have closer contact with the river in their daily activities. In line, a recent study conducted in Kaédi by Gbalégba and colleagues identified 12 types of water contact activities that populations living close to the dam take part in, including swimming/bathing (*n* = 3788, 36.9%); washing clothes (*n* = 2016, 19.7%); and washing dishes (*n* = 1322, 12.9%) [31].

The knowledge of the aetiology of schistosomiasis is influenced by socio-cultural beliefs. In the current study, populations in both localities did not know the transmission pathway well. Data also show that the education level has no influence on the respondents’ knowledge in Korhogo. In fact, the knowledge of disease aetiology was related to drinking unsafe water, environmental factors (sun and unsafe water), and mysticism. Drinking unsafe water was perceived to be the major cause of infection [32]. The respondents argued that unsafe drinking water or practising lugubrious activities, as well as exposure to sunrays, leads to infection. The study furthermore shows that being contaminated with urine of animals such dogs and goats is perceived as a being the main source of transmission. This perception might be due to the low transmission levels of the disease in the study areas. Nonetheless, a study conducted in an endemic zone of schistosomiasis transmission in Niger [29] revealed that the community’s ignorance of the disease put it at a higher risk of infection. It appears that living in an endemic area is not a sufficient condition for knowing pathology.

In Kaédi, the aetiology of the disease was unknown, while the symptoms of the urinary form were well known. This variation of schistosomiasis knowledge was also observed in a study done in Nampula province, Mozambique [28], where the community knew the symptoms of schistosomiasis but lacked the knowledge on the causes, putting them in a position of vulnerability. The lack of knowledge of the aetiology observed in Korhogo and Kaédi could be attributable to the absence of sensitisation activities in both localities. In fact, a study conducted in Niger demonstrated that at the beginning of the study schistosomiasis was little known, but that participants’ knowledge improved moderately after one year of sensitisation [29]. Hence, health education may be an important means of improving individual recognition of the disease [33].

The study also showed that knowledge of prevention measures was low and that a person’s education level impacts weakly on this knowledge. In Kaédi, the education level influenced the knowledge of schistosomiasis prevention measures. In fact, the social construction of people, i.e., how people perceive the transmission of the disease in their socio-cultural context appeared to be important factors of exposure to infection. Thus, knowledge of prevention measures is influenced by knowledge of disease causes. The study also showed that in Korhogo the population did not use any schistosomiasis prevention strategies. It is therefore clear that a lack of knowledge of schistosomiasis in general influences the choice of treatment and, more importantly, people’s attitude to the disease. In Yemen, it was observed that a lack of knowledge of the disease influenced the knowledge of prevention means. In Sri Lanka, care-seeking behaviours of mothers and maternal knowledge of symptoms contributed to reducing childhood disease [34]. Hence, the lack of knowledge of prevention measures exposes individuals to a risk of infection. In addition, the respondents mentioned that the ‘risky’ way they live in connection with their immediate environment prevents them to protect themselves against schistosomiasis. The same finding has been reported elsewhere. Communities around Lake Victoria in Uganda and Kenya, for instance, know how to avoid the disease, but they state that there is nothing they can do due to their dependency on water for domestic and economic use [35, 36]. In line with a lack of knowledge concerning prevention means, previous studies in Brazil and Egypt have also demonstrated that avoiding drinking unsafe water is perceived as an effective means to prevent infection [32, 37].

Our work furthermore shows that care practices varied between ethnic groups. The knowledge of the disease and the social construction around it influences people’s care-seeking choices. This leads to people using methods that are in contrast to those advocated by biomedical sciences and which may result in a delay in effective healthcare. The same result has been found by Mandelzweig and colleagues who show that perceptual, social and behavioural factors contribute to a delay in seeking medical care in acute ischemic cases [38]. Along the same lines, many authors argue that health beliefs are important barriers to care seeking [34, 39, 40].

It was found that using various herbal remedies to treat schistosomiasis is practised by people in both communities. However, this often prevents people from seeking medical treatment [41]. The absence of good care practices is reflected in the fact that communities often do not know that there are medical means to manage the disease. Beyond this, there are environmental and social factors that influence the search for care. This observation was made by MacKian in his study on health systems. He showed that social, environmental and economic factors influence communities’ healthcare-seeking behaviours [42]. The lack of knowledge due to a weak prevalence of schistosomiasis in both study sites leads to communities relying on means that maintain and aggravate the disease [43]. However, a study conducted in the Magu district of Tanzania observed that in endemic areas patients refer to traditional healers as a care-seeking behaviour [44]. As it is noticed that people did not know the disease, their seeking treatment pathway totally differed from the biomedical’s, hence they were in a position of vulnerability with regards to schistosomiasis.

## Conclusions

This study aimed to add to the limited literature exploring KAP related to schistosomiasis control at the community and household levels. The study showed that schistosomiasis is poorly known in both West African settings assessed. It was found that communities tended to rely on traditional cures, and that persisting misconceptions pose barriers to effective disease prevention and control. In addition, there is little knowledge on how to prevent schistosomiasis and the causes of disease. It is therefore important to sensitise communities about schistosomiasis treatment and its efficacy. Policymakers and health organisations should contribute to improve knowledge at the individual and community levels. This could be made possible through a participative or integrated approach to the disease by combining treatments with community health education at the grassroots level.

## Additional files

Additional file 1: Multilingual abstracts in the six official working languages of the United Nations. (PDF 591 kb)

Additional file 2: **Table S1.** Sociodemographic characteristics of informants. (DOC 58 kb)

## Abbreviations

FGD: Focus group discussion
KAP: Knowledge, attitudes and practices
NGO: Non-governmental organisation
