# Correction to: Community knowledge, attitudes and practices related to schistosomiasis and associated healthcare-seeking behaviours in northern Côte d’Ivoire and southern Mauritania

**DOI:** 10.1186/s40249-018-0476-6

**Published:** 2018-08-23

**Authors:** Amoin Jeanne d’Arc Koffi, Mohamed Doumbia, Gilbert Fokou, Moussa Keita, Brama Koné, N’doumy Noel Abé

**Affiliations:** 1 Department of Research and Development, Centre Suisse de Recherches Scientifiques en Côte d’Ivoire, Abidjan, Côte d’Ivoire; 2 Environment and Communication Training Unit, University Alassane Ouattara, Bouaké, Côte d’Ivoire; 3 Ethno-Sociology Institute, University Félix Houphouët-Boigny, Abidjan, Côte d’Ivoire; 4 University of Nouakchott, Nouakchott, Mauritania; 5 Institute of Agro-Pastoral Management, University Péléforo-Gbon-Coulibaly, Korhogo, Côte d’Ivoire

## Correction

After publication of this article [1] it came to our attention that Tables 2, 3, 4, 5, 6, 7 were presented incorrectly. The correct tables can be found below and the table citations in the article were corrected as well. The original article has been updated to reflect this change.

**Table 2:** Knowledge of schistosomiasis symptoms according to locality*

	Kaédi *n* = 503		Korhogo *n* = 195		Total *n* = 698	
	Yes (*n*)	Yes (%)	Yes (*n*)	Yes (%)	Yes (*n*)	Yes (%)
*Do you know about schistosomiasis symptoms?*	441	87.7	143	73.3	584	83.7
Pain during urination	154	34.9	42	29.4	196	33.6
Low urine output	194	44.0	10	7.0	204	34.9
Blood in stool	204	46.3	72	50.3	276	47.3
Stomach pain	93	21.1	8	5.6	101	17.3
a Headache	61	13.8	7	4.9	68	11.6
Blood in urine	382	86.6	101	70.6	483	82.7
^a^Other symptoms	14	3.2	25	17.5	39	6.7

*Multiple choice question;^a^ Wound on genitals, constipation; *n*: Number of respondents

**Table 3:** Population’s knowledge of schistosomiasis symptoms according to level of literacy in Korhogo and Kaédi

	No education (%)	Arabic (%)	Primary (%)	Secondary (%)	High school (%)	*P*-value
Korhogo	*n* = 576	*n* = 155	*n* = 263	*n* = 343	*n* = 107	
*Knowledge of schistosomiasis*	54 (9.4)	17 (11)	49 (18.6)	106 (30.6)	45 (42.1)	**≤ 0.001** ^**a**^
*Knowledge of symptom* ^**d**^	23 (42.6)	11 (64.7)	24 (49.0)	54 (50.9)	29 (64.4)	0.856^a^
Pain during urination	9 (39.1)	6 (54.4)	9 (37.5)	14 (24.6)	4 (0.05)	0.5^b^
Low urine output	3 (13.0)	1 (9.1)	3 (12.5)	3 (5.3)	0 (0.0)	N/A
Blood in stool	11 (47.8)	4 (36.4)	8 (33.3)	31 (54.4)	16 (55.2)	0.384^a^
Stomach pain	0 (0.0)	1 (9.1)	2 (8.2)	3 (5.3)	1 (3.4)	N/A
Headache	0 (0.0)	0 (0.0)	1 (4.2)	4 (7.0)	2 (6.9)	N/A
Blood in urine	11 (47.8)	9 (81.8)	19 (79.2)	39 (68.4)	22 (75.9)	0.111^a^
^c^Other symptoms	7 (30.4)	2 (18.2)	1 (4.2)	8 (14.0)	7 (24.1)	0.135^b^
Kaédi	*n* = 473	*n* = 472	*n* = 236	*n* = 195	*n* = 72	
*Knowledge of schistosomiasis*	276 (58.4)	228 (48.3)	122 (51.7)	107 (54.9)	35 (48.6)	**0.031** ^**a**^
*Knowledge of symptom* ^**d**^	170 (61.6)	114 (50.0)	67 (54.9)	66 (61.7)	23 (65.7)	0.441^a^
Pain during urination	52 (30.6)	49 (43.0)	24 (35.8)	18 (27.3)	10 (43.5)	0.129^a^
Low urine output	66 (38.8)	65 (57.0)	26 (38.8)	21 (31.8)	15 (65.2)	**0.001** ^**a**^
Blood in stool	76 (44.7)	56 (49.1)	29 (43.3)	29 (43.9)	13 (56.5)	0.755^a^
Stomach pain	35 (20.6)	30 (26.3)	12 (17.9)	8 (12.1)	7 (30.4)	0.151^a^
Headache	28 (16.5)	22 (19.3)	6 (9.0)	2 (3.0)	3 (13.0)	**0.020** ^**b**^
Blood in urine	144 (84.7)	98 (86.0)	61 (91.0)	61 (92.4)	17 (73.9)	0.146^a^
^c^Other symptoms	8 (4.7)	3 (2.6)	3 (4.5)	0 (0.0)	0 (0.0)	N/A

Significant *P*-values (< 0.05) are highlighted in bold

^a^*P*-value based on chi-square test; ^b^*P*-value based on Fisher’s exact test; ^c^Wound on the reproductive organs, constipation; ^**d**^ Multiple choice question; *n*: Number of respondents

**Table 4:** Knowledge of transmission pathway of schistosomiasis according to locality*

	Kaédi *n* = 779		Korhogo *n* = 289		Total *n* = 1068	
	Yes (*n*)	Yes (%)	Yes (*n*)	Yes (*n*)	Yes (%)	Yes (%)
*Do you know how schistosomiasis is transmitted?*	497	63.8	188	65.1	685	64.1
Drinking unsafe water	312	62.8	72	38.3	384	56.1
Eating poorly washed fruits, vegetables	81	16.3	5	2.7	86	12.6
Swimming in rivers, drains, etc.	295	59.4	124	66.0	419	61.2
Contact with infected person	41	8.2	15	8.0	56	8.2
Walking barefoot on infected urine	110	22.1	13	6.9	123	18.0
Contact with dirty water	253	50.9	81	43.1	334	48.8
^a^Other transmission	38	7.6	22	11.7	60	8.8
Do not know	6	1.2	2	1.1	8	1.2

*Multiple choice question; *n*: Number of respondents; ^a^ Avoiding contact with goat urine, wearing protective shoes, avoiding drinking hot water

**Table 5:** Knowledge of transmission pathways of schistosomiasis according to level of literacy in Korhogo and Kaédi*

	No education (%)	Arabic (%)	Primary (%)	Secondary (%)	High school (%)	*P*-value
Korhogo	*n* = 26	*n* = 13	*n* = 33	*n* = 75	*n* = 38	
Drinking unsafe water	8 (29.6)	5 (38.5)	11 (34.4)	32 (42.1)	15 (39.5)	0.817^b^
Eating poorly washed fruits, vegetables	1 (3.7)	1 (7.7)	0 (0.0)	2 (2.6)	1 (2.6)	N/A
Swimming in rivers, drains, etc.	11 (40.7)	7 (53.8)	25 (78.1)	51 (67.1)	26 (68.4)	0.035^a^
Contact with infected person	4 (14.8)	3 (23.1)	1 (3.1)	4 (5.3)	1 (2.6)	N/A
Walking barefoot on infected urine	3 (11.1)	1 (7.7)	1 (3.1)	8 (10.59	N/A	0.217^b^
Contact with dirty water	11 (40.7)	9 (69.2)	10 (31.3)	35 (46.1)	15 (39.5)	0.200^a^
^c^Other	6 (22.2)	2 (15.4)	2 (6.3)	10 (13.2)	2 (5.3)	0.230^b^
Do not know	1 (3.7)	0 (0.0)	0 (0.0)	0 (0.0)	1 (2.6)	N/A
Kaédi	*n* = 189	*n* = 136	*n* = 86	*n* = 76	*n* = 27	
Drinking unsafe water	137 (72.5)	76 (60.3)	44 (55.0)	36 (47.4)	18 (66.7)	**0.001** ^**a**^
Eating poorly washed fruits, vegetables	26 (13.8)	29 (23.0)	13 (16.3)	7 (9.2)	6 (22.2)	0.073^a^
Swimming in rivers, drains, etc.	79 (41.8)	82 (65.1)	56 (70.0)	59 (77.6)	18 (66.7)	**< 0.001** ^a^
Contact with infected person	9 (4.8)	13 (10.3)	9 (11.3)	5 (6.6)	5 (19.2)	0.058^a^
Walking barefoot on infected urine	45 (23.8)	31 (24.6)	13 (16.3)	9 (11.8)	12 (44.4)	**0.005** ^**a**^
Contact with dirty water	85 (45.0)	73 (57.9)	42 (52.5)	36 (47.4)	16 (59.3)	0.175^a^
^c^Other	17 (9.0)	13 (10.3)	3 (3.8)	3 (3.9)	2 (7.4)	0.291^b^
Do not know	3 (1.6)	1 (0.8)	0 (0.0)	2 (2.6)	0 (0.0)	N/A

Statistically significant *P*-values (< 0.05) are highlighted in bold

***n***: Number of respondents; ^a^*P*-value based on chi-square test; ^b^*P*-value based on Fisher’s exact test; ^c^Contact with goat urine, not wearing protective shoes, drinking hot water, exposure to sunlight; *Multiple choice question

**Table 6:** Knowledge of schistosomiasis prevention measures according to locality*

	Kaédi *n* = 1450		Korhogo *n* = 1443		Total *n* = 2893	
	Yes (*n*)	Yes (%)	Yes (*n*)	Yes (%)	Yes (*n*)	Yes (%)
*Do you know how to avoid schistosomiasis?*	438	30.2	173	12.0	611	21.1
Avoid eating certain foods	77	17.6	4	2.3	81	13.3
Avoid drinking unsafe water	285	65.1	86	49.7	371	60.7
Avoid swimming in rivers, drains, etc.	214	48.9	132	76.3	346	56.6
Avoid urinating in water bodies	198	45.2	37	21.4	235	38.5
Avoid defecating in water bodies	201	45.9	31	17.9	232	38.0
^a^Other	25	5.7	24	13.9	49	8.0
Do not know	6	1.4	2	1.2	8	1.3

*Multiple choice question; *n*: Number of respondents; ^a^ Avoid contact with goat urine, wear protective shoes, avoid drinking hot water

**Table 7:** Knowledge of schistosomiasis prevention measures according to level of literacy in Korhogo and Kaédi*

	No education (%)	Arabic (%)	Primary (%)	Secondary (%)	High school (%)	*P*-value
Korhogo	*n* = 23	*n* = 14	*n* = 34	*n* = 68	*n* = 33	
Avoid eating certain foods	0 (0.0)	2 (14.3)	1 (2.9)	1 (1.5)	0 (0.0)	N/A
Avoid drinking unsafe water	9 (39.1)	5 (35.7)	16 (47.1)	39 (57.4)	16 (48.5)	0.430^b^
Avoid swimming in rivers, drains, etc.	11 (47.8)	6 (42.9)	27 (79.4)	60 (88.2)	26 (78.8)	**< 0.001** ^**a**^
Avoid urinating in water bodies	6 (26.1)	4 (28.6)	3 (8.8)	17 (25.0)	6 (18.2)	0.319^b^
Avoid defecating in water bodies	3 (13.0)	1 (7.1)	2 (5.9)	18 (26.5)	6 (18.2)	0.079^b^
^a^Other	5 (21.7)	5 (35.7)	3 (8.8)	7 (10.3)	4 (12.1)	0.082^b^
Do not know	0 (0.0)	0 (0.0)	0 (0.0)	2 (2.9)	0 (0.0)	N/A
Kaédi	*n* = 174	*n* = 106	*n* = 63	*n* = 72	*n* = 24	
Avoid eating certain foods	30 (17.2)	25 (23.6)	11 (17.5)	5 (6.9)	6 (25.0)	0.056^b^
Avoid drinking unsafe water	135 (77.6)	63 (59.4)	35 (55.6)	36 (50.7)	16 (66.7)	**< 0.001** ^**a**^
Avoid swimming in rivers, drains, etc.	54 (31.0)	58 (54.7)	38 (60.3)	50 (70.4)	14 (58.3)	**< 0.001** ^**a**^
Avoid urinating in water bodies	76 (43.7)	60 (56.6)	24 (38.1)	26 (36.6)	12 (50.0)	0.053^a^
Avoid defecating in water bodies	77 (44.3)	60 (56.6)	25 (39.7)	27 (38.0)	12 (50.0)	0.092^a^
^c^Other	8 (4.6)	8 (7.5)	5 (7.9)	2 (2.8)	2 (8.3)	0.548^b^
Do not know	2 (1.2)	2 (1.9)	2 (3.2)	0 (0.0)	0 (0.0)	N/A

Statistically significant *P*-values (< 0.05) are highlighted in bold

***n***: number of respondents; ^a^*P*-value based on chi-square test; ^b^*P*-value based on Fisher’s exact test; ^c^avoiding contact with goat urine, wearing protective shoes, avoiding drinking hot water; *Multiple choice question
