# Supplementary material for: Community knowledge, attitudes and practices related to schistosomiasis and associated healthcare-seeking behaviours in northern Côte d’Ivoire and southern Mauritania
Source: Infect Dis Poverty. 2018 Jul 10;7:70. doi: 10.1186/s40249-018-0453-0 (PMC6038328; doi:10.1186/s40249-018-0453-0)
Supplement: Supplementary file 1 — Multilingual abstracts in the six official working languages of the United Nations. (PDF 591 kb) [file 40249_2018_453_MOESM1_ESM.pdf]

Translation of the abstract into the six official working languages of the United Nations

المعرفة المجتمعية والأوضاع والممارسات المتعلقة بالبلهارسيا، والسلوكيات المرتبطة بها في مجال الرعاية الصحية في شمال كوت ديفوار وجنوب موريتانيا.

أموان جان دارك كوفي، محمد دومبيا، جلبرت فوكو، موسى كيتا، براما كونى، ندومي نويل آبي

الملخص.

الخلفية: من بين الأمراض الطفيلية، تحتل البلهارسيا المرتبة الثانية من حيث نسبة انتشارالمرض العالمية بعد الملاريا. ورغم الجهود المبذولة للحد من انتقال المرض، يوجد أكثر من 230 مليون مصاب، يعيش 85% منهم في أفريقيا جنوب الصحراء الكبرى. وبينما دُرِست الخصائص الوبائية للبلهارسيا على نطاق واسع عبر المناطق الموبوءة، إلا أن العوامل الاجتماعية قد حظيت باهتمام أقل. تقوم الدراسة الحالية بتقييم المعرفة المجتمعية لأسباب البلهارسيا ، وانتقالها ، وعلاماتها ، وأعراضها ، والوقاية منها ، بالإضافة إلى سلوكيات البحث عن الرعاية الصحية في موضعين من غرب أفريقيا ، بهدف تعزيز تدخلات مكافحة البلهارسيا.

الطرق: أجرينا استقصائيين مقطعيين في كورهوجو بكوت ديفوار، وكيهيدي بموريتانيا من أغسطس 2014 إلى يونيه 2015. وقمنا بعمل استبيان لجمع بيانات كميّة على مستوى الأسرة في كورهوجو (حجم العينة = 1456) وفي كيهيدي (حجم العينة = 1453). مناقشات مجموعة التركيز (كورهوجو: حجم العينة = 32 ، كيهيدي: حج العينة = 32) والتصوير التشاركي (صوت الصورة photovoice) كورهوجو :حجم العينة = 16، كيهيدي: حجم العينة = 16) أُجريت داخل المجتمعات المحلية لجمع البيانات النوعية. وعلاوة على ذلك، استخدمت مقابلات شبه منظمة للنقاش مع مقدمي المعلومات الرئيسيين من برامج المكافحة والمنظمات غير الحكومية و المناطق الصحية (كورهوجو: حجم العينة = 8، كيهيدي: حجم العينة = 7).

النتائج: أظهرت الدراسة أن مرض البلهارسيا غير معروف بشكل جيد لدى المجتمعات المحلية، وقد زعم 64.1% أنهم يعرفون أسباب المرض، لكن الحقيقة مختلفة. وتأتي هذه المعرفة من مصدر ثقافي أكثر منها من مصدر طبي حيوي. وقد لوحظ أن التركيب الإجتماعي للمرض مختلف عن التعريف الطبي الحيوي. في كورهوجو، كانت البلهارسيا غالباً مرتبطة بالعديد من الأمراض الأخرى، لاسيما قرحة المعدة والسيلان. ويؤمن السكان أن البلهارسيا تنتج عن التعرض لبول الماعز أو بول الكلاب في البيئة المحيطة. في كيهيدي، يُنظَرإلى البلهارسيا كمرض انتقل بواسطة عناصر بيئية مثل أشعة الشمس والمياه الملوثة . وفي كلا الموضعين، وُجد أن سبل التماس الرعاية تأثرت بشكل كبير بالعادات المحلية والمعالجة الذاتية المكتسبة من القطاع غير الرسمي.

الاستنتاجات: كشفت هذه الدراسة أن المعرفة بشأن مسببات البلهارسيا وانتقالها وأعراضها والوقاية منها وعلاجها بين السكان في كورهوجو وكيهيدي، مرتكزة على ثقافتهم المحلية. ولذلك يمكن أن تشكل العادات المتأصلة عقبة كبيرة في التخلص من البلهارسيا.

Translated from English version into Arabic by Susan Fegy, proofread by Salma Madani, through

TRANSLATORS
WITHOUT BORDERS

科特迪瓦北部和毛里塔尼亚南部关于血吸虫病的社区认知、态度和行为及相关的求医行为

Amoin Jeanne d'Arc Koffi, Mohamed Doumbia, Gilbert Fokou, Moussa Keita, Brama Koné and N'doumy Noel Abé

摘要：

引言：在寄生虫感染中，血吸虫病在世界范围内发病率仅次于疟疾。尽管采取了相关控制措施，仍有超过 2.3 亿人感染，其中 85%生活在撒哈拉以南非洲地区。尽管对血吸虫病的流行特征进

行了广泛研究，然而社会学因素却很少受到关注。本研究评估了两个西非地区涉及血吸虫病的病因、传播、体征、症状和预防的社区知识及求医行为，以加强对血吸虫病的控制干预。
方法：2014 年 8 月至 2015 年 6 月，我们在科特迪瓦的科霍戈和毛里塔尼亚的卡埃迪进行了两项横断面调查。使用调查问卷收集家庭层面（科霍戈 1456 份、卡埃迪 1453 份）的定量数据。在社区内进行焦点小组讨论（科霍戈 32 份、卡埃迪 32 份）和参与式摄影即影像发声法(photovoice)（科霍戈 16 份、卡埃迪 16 份），收集定性材料。此外，采用半结构式访谈与控制项目、非政府组织和卫生区的关键信息提供者进行讨论（科霍戈 8 份、卡埃迪 7 份）。
结果：研究表明，血吸虫病并不为社区居民所熟知，64.1%的被访者声称了解其病因，但实际并非如此。这些知识多来自其文化而非生物医学领域。据观察，该疾病的社会认知与生物医学定义不同。在科霍戈，血吸虫病通常被认为与其他几种疾病相关，尤其是胃溃疡和淋病。人们相信血吸虫病是与山羊或狗尿液接触所致，在卡埃迪，血吸虫病被认为是由环境因素，如通过阳光和污水传播。在这两种情况下，当地习俗和非正式部门提供的自我用药严重影响了患者的求医行为。
结论：本研究揭示了科霍戈和卡埃迪人群对血吸虫病的病因学、传播、症状、阻断和治疗的认知主要是基于当地文化。因此，根深蒂固的习惯可能是消除血吸虫病的重大障碍。

Translated from English version into Chinese by Translated by Peng Song, edited by Pin Yang

CHINESE CENTER FOR DISEASE CONTROL AND PREVENTION
NATIONAL INSTITUTE OF PARASITIC DISEASES

Connaissances, attitudes et pratiques communautaires liées à la schistosomiase et aux comportements associés à la recherche de soins de santé dans le nord de la Côte d'Ivoire et le sud de la Mauritanie

Amoin Jeanne d'Arc Koffi, Mohamed Doumbia, Gilbert Fokou, Moussa Keita, Brama Koné et N'doumy Noel Abé

Résumé

Contexte: Parmi les infections parasitaires, la schistosomiase occupe la deuxième place après le paludisme en termes de morbidité mondiale. Malgré les efforts pour contenir sa transmission, plus de 230 millions de personnes sont infestées, dont 85 % vivent en Afrique subsaharienne. Alors que les caractéristiques épidémiologiques de la schistosomiase ont été largement étudiées dans des contextes endémiques, les facteurs sociaux ont été moins pris en compte. La présente étude évalue les connaissances communautaires sur les causes, la transmission, les signes, les symptômes et la prévention de la schistosomiase, ainsi que les comportements de recherche de soins dans deux localités d'Afrique de l'Ouest, dans le but de renforcer les interventions de lutte contre la schistosomiase.
Méthodes: D'août 2014 à juin 2015, nous avons mené deux enquêtes transversales à Korhogo, en Côte d'Ivoire, et à Kaédi, en Mauritanie. Nous avons utilisé un questionnaire pour collecter des données quantitatives auprès des ménages de Korhogo ($n = 1456$) et Kaédi ($n = 1453$). Des groupes de discussion (Korhogo : $n = 32$, Kaédi : $n = 32$) et des enquêtes participatives utilisant la photographie (Photovoice ; Korhogo : $n = 16$, Kaédi : $n = 16$) ont été organisés au sein des communautés afin de recueillir des données qualitatives. En outre, des entretiens semi-structurés ont été utilisés pour discuter

avec des informateurs clés dans les programmes de lutte, les organisations non gouvernementales et les districts sanitaires (Korhogo : *n* = 8, Kaédi : *n* = 7).

Résultats: L'étude a démontré que la schistosomiase était mal connue dans les communautés; 64,1 % des personnes interrogées ont affirmé connaître les causes de la maladie, mais la réalité est différente. Cette connaissance est plus d'origine culturelle que biomédicale. Nous avons observé que la construction sociale de la maladie était différente de la définition biomédicale. À Korhogo, la schistosomiase était souvent associée à plusieurs autres maladies, notamment l'ulcère de l'estomac et la gonorrhée. La population croit que la schistosomiase est causée par l'exposition à l'urine de chèvre ou de chien dans l'environnement. À Kaédi, la schistosomiase est considérée comme une maladie transmise par des éléments environnementaux tels que le soleil et l'eau sale. Dans ces deux contextes, les parcours de recherche de soins sont fortement influencés par les coutumes locales et par l'automédication avec des médicaments achetés dans le secteur informel.

Conclusions: Cette étude a révélé que les connaissances sur l'étiologie, la transmission, les symptômes, la prévention et le traitement de la schistosomiase parmi les populations de Korhogo et Kaédi étaient fondées sur leur culture locale. Des habitudes profondément enracinées pourraient donc constituer un obstacle important à l'élimination de la schistosomiase.

Translated from English version into French by Adole Simon, proofread by Suzanne Assenat, through

TRANSLATORS
WITHOUT BORDERS

Общинные знания, отношение и практический подход к шистосомозу и связанные с ним особенности поведения при обращении за медицинской помощью в северной части Кот-д'Ивуара и в южной Мавритании.

Амуан Жанн д'Арк Коффи, Мохамед Думбия, Жильбер Фоку, Мусса Кейта, Брама Конэ и Н'думи Ноэль Абэ

Аннотация

Краткое описание: Среди паразитарных инфекций шистосомоз занимает второе место после малярии по смертности во всём мире. Несмотря на усилия по сдерживанию передачи инфекции, заражено более 230 миллионов людей, из которых 85% проживают в Африке к югу от Сахары. В то время, как эпидемиологические характеристики шистосомоза всесторонне изучены в различных эндемических средах, социальным факторам уделялось меньше внимания. В целях усиления мер по борьбе с шистосомозом в настоящем исследовании сделана оценка общинных знаний о случаях заболевания шистосомозом, способах его передачи, признаках, симптомах и методах предотвращения заражения, а также об особенностях поведения при обращении за медицинской помощью в двух регионах Западной Африки.

Методы: С августа 2014 года по июнь 2015 года мы провели два перекрёстных обследования в городах Корхого, Кот-д'Ивуар, и Каэди, Мавритания. Для сбора количественных данных на

уровне домохозяйств в Корхого (n = 1456) и Каэди (n = 1453) мы использовали опросную анкету. Для получения качественных данных среди общин были организованы дискуссии фокус-групп (Корхого: n = 32, Каэди: n = 32) и применён метод фотосъёмки с активным вовлечением участников опроса (PhotoVoice) (Корхого: n = 16, Каэди: n = 16). Кроме того, были проведены полуформализованные интервью с главными опрашиваемыми представителями программ по борьбе с болезнью, неправительственных организаций и здравоохранительных округов (Корхого: n = 8, Каэди: n = 7).

Результаты: Исследование показало, что в общинах мало знают о шистосомозе; 64,1% опрошенных утверждали, что им известны причины болезни, однако в действительности это не так. Их знания происходили в основном из культурного источника, а не из биомедицинского. Было замечено, что социальное конструирование болезни отличается от биомедицинского определения. В Корхого шистосомоз часто ассоциировался с несколькими другими болезнями, в частности, с язвой желудка и гонореей. Население верит, что причиной шистосомоза является контакт с козлиной или собачьей мочой в окружающей среде. В Каэди шистосомоз считают болезнью, которая переносится элементами окружающей среды, такими как солнечный свет и грязная вода. В обоих регионах было установлено, что способы получения помощи сильно зависят от местных обычаев и методов самолечения, полученных неформальным путём.

Выводы: Настоящее исследование выявило, что знания об этиологии, передаче, симптомах, предотвращении и лечении шистосомоза у населения в Корхого и Каэди основаны на местной культуре. Следовательно, глубоко укоренившиеся привычки могут послужить серьёзным препятствием при устранении шистосомоза.

Translated from English version into Russian by Natalia Potashnik, proofread by Liudmila Tomanek, through

TRANSLATORS WITHOUT BORDERS

Conocimientos, actitudes y prácticas de la comunidad en relación con la esquistosomosis y los comportamientos asociados en la búsqueda de atención sanitaria en el norte de Costa de Marfil y el sur de Mauritania

Amoin Jeanne d'Arc Koffi, Mohamed Doumbia, Gilbert Fokou, Moussa Keita, Brama Koné y N' doumy Noel Abé

Resumen

Antecedentes: Entre las infecciones parasitarias, la esquistosomosis ocupa el segundo lugar después de la malaria en términos de morbilidad mundial. A pesar de los esfuerzos por controlar la transmisión, más de 230 millones de personas están infectadas, de las que el 85 % viven en África Subsahariana. Mientras las características epidemiológicas de la esquistosomosis han sido ampliamente estudiadas en entornos endémicos, se ha prestado menos atención a los factores sociales. El presente estudio evalúa el

conocimiento de la comunidad sobre las causas de la esquistosomosis, su transmisión, signos, síntomas y prevención, así como las conductas de búsqueda sanitarias en dos entornos de África Occidental, con el objetivo de fortalecer las intervenciones para el control de la esquistosomosis.

Métodos: Desde agosto de 2014 hasta junio de 2015, realizamos dos encuestas transversales en Korhogo, Costa de Marfil, y en Kaédi, Mauritania. Utilizamos un cuestionario para recopilar datos cuantitativos a nivel de hogar en Korhogo (n = 1456) y en Kaédi (n = 1453). Para recoger datos cualitativos en las comunidades, se llevaron a cabo debates de grupos específicos (Korhogo: n = 32, Kaédi: n = 32) y fotografía participativa (photovoice) (Korhogo: n = 16, Kaédi: n = 16). Además, se utilizaron entrevistas semiestructuradas para debatir con informadores clave sobre los programas de control, organizaciones no gubernamentales y distritos sanitarios (Korhogo: n = 8, Kaédi: n = 7).

Resultados: El estudio demostró que la esquistosomosis no se conoce bien en las comunidades; el 64,1 % afirmó saber las causas de la enfermedad, pero la realidad es distinta. Este conocimiento proviene más de una fuente cultural que biomédica. Se observó que la construcción social de la enfermedad es diferente de la definición biomédica. En Korhogo, la esquistosomiasis se asociaba frecuentemente con otras enfermedades, sobre todo úlcera estomacal y gonorrea. La población cree que la causa de la esquistosomiasis es la exposición a la orina de cabra o de perro en el medio ambiente. En Kaédi la esquistosomiasis se considera una enfermedad transmitida por elementos del medio ambiente como la luz solar o el agua sucia. En ambos escenarios, se constató que los protocolos de atención médica estaban fuertemente influenciados por las costumbres locales y la medicación sin supervisión desde el sector informal.

Conclusiones: Este estudio reveló que el conocimiento de la etiología, transmisión, síntomas, prevención y tratamiento de la esquistosomiasis entre los habitantes de Korhogo y Kaédi se basa en su cultura local. Por lo tanto, los hábitos profundamente arraigados podrían constituir un obstáculo importante para la eliminación de la esquistosomiasis.

Translated from English version into Spanish by Iria Carballo, proofread by María Florencia Paolillo, through

TRANSLATORS
WITHOUT BORDERS
