# Supplementary material for: Community knowledge, attitudes and practices related to schistosomiasis and associated healthcare-seeking behaviours in northern Côte d’Ivoire and southern Mauritania
Source: Infect Dis Poverty. 2018 Jul 10;7:70. doi: 10.1186/s40249-018-0453-0 (PMC6038328; doi:10.1186/s40249-018-0453-0)
Supplement: Supplementary file 2 — Table S1. Sociodemographic characteristics of informants. (DOC 58 kb) [file 40249_2018_453_MOESM2_ESM.doc]

**Table S1 Sociodemographic characteristics of informants**

|  | Kaédi | Korhogo |  |
| --- | --- | --- | --- |
|  | n=1453 (%) | n=1456 (%) | *P*-value |
| **Gender** |  |  | 0.981 |
| Male | 661 (45.5) | 663 (45.5) |  |
| Female | 792 (54.5) | 793 (54.5) |  |
| Total | 1453 (100.0) | 1456 (100.0) |  |
| **Marital status** |  |  | **0.000** |
| Single | 107 (7.4) | 326 (22.5) |  |
| In monogamous marriage | 952 (65.6) | 713 (49.1) |  |
| In polygamous marriage | 182 (12.5) | 210 (14.5) |  |
| Divorced/separated | 49 (3.4) | 14 (1.0) |  |
| Widower/widow | 151 (10.4) | 117 (8.1) |  |
| Non-married partnership | 11 (0.8) | 71 (4.9) |  |
| Total | 1452 (100.0) | 1451 (100.0) |  |
| **RELIGION** |  |  | **0.000** |
| Christians | 19 (1.3) | 305 (21.0) |  |
| Muslims | 1423 (98.0) | 1039 (71.5) |  |
| Animists | 9 (0.6) | 47 (3.2) |  |
| None | 1 (0.1) | 57 (3.9) |  |
| Others | 0 (0.0) | 6 (0.4) |  |
| Total | 1452 (100.0) | 1454 (100.0) |  |
| **Occupation** |  |  | **0.000** |
| Farmer-herdsman | 204 (14.1) | 104 (7.3) |  |
| Trader, artisan | 440 (30.5) | 596 (41.6) |  |
| Teacher | 62 (4.3) | 63 (4.4) |  |
| Medical officer | 19 (1.3) | 26 (1.8) |  |
| Manager, Engineer, Technician and Office Clerk | 93 (6.4) |  |  |
| Other activities | 255 (17.7) | 279 (19.5) |  |
| Not applicable | 7 (0.5) | 2 (0.1) |  |
| None | 122 (8.5) | 57 (4.0) |  |
| Undeclared | 59 (4.1) | 6 (0.4) |  |
| Household | 76 (5.3) | 57 (4.0) |  |
| Student | 4 (0.3) | 20 (1.4) |  |
| Retired | 102 (7.1) | 82 (5.7) |  |
| Total | 1443 (100.0) | 1434 (100.0) |  |
| **Education Level** |  |  | **0.000** |
| No education | 474 (32.7) | 576 (39.8) |  |
| Arabic school | 472 (32.6) | 155 (10.7) |  |
| Primary school | 236 (16.3) | 265 (18.3) |  |
| Secondary school | 196 (13.5) | 343 (23.7) |  |
| High school | 72 (5.0) | 107 (7.4) |  |
| Total | 1450 (100.0) | 1446 (100.0) |  |
